# Impact of cancer-associated fibroblasts on survival of patients with ampullary carcinoma

**DOI:** 10.3389/fonc.2023.1072106

**Published:** 2023-03-16

**Authors:** Kosei Takagi, Kazuhiro Noma, Yasuo Nagai, Satoru Kikuchi, Yuzo Umeda, Ryuichi Yoshida, Tomokazu Fuji, Kazuya Yasui, Takehiro Tanaka, Hajime Kashima, Takahito Yagi, Toshiyoshi Fujiwara

**Affiliations:** ^1^ Department of Gastroenterological Surgery, Okayama University Graduate School of Medicine, Dentistry, and Pharmaceutical Sciences, Okayama, Japan; ^2^ Department of Pathology, Okayama University Graduate School of Medicine, Dentistry, and Pharmaceutical Sciences, Okayama, Japan

**Keywords:** ampullary carcinoma, carcinomas of the papilla of Vater, cancer-associated fibroblast, outcome, survival, recurrence

## Abstract

**Background:**

Cancer-associated fibroblasts (CAFs) reportedly enhance the progression of gastrointestinal surgery; however, the role of CAFs in ampullary carcinomas remains poorly examined. This study aimed to investigate the effect of CAFs on the survival of patients with ampullary carcinoma.

**Materials and methods:**

A retrospective analysis of 67 patients who underwent pancreatoduodenectomy between January 2000 and December 2021 was performed. CAFs were defined as spindle-shaped cells that expressed α-smooth muscle actin (α-SMA) and fibroblast activation protein (FAP). The impact of CAFs on survival, including recurrence-free (RFS) and disease-specific survival (DSS), as well as prognostic factors associated with survival, was analyzed.

**Results:**

The high-α-SMA group had significantly worse 5-year RFS (47.6% vs. 82.2%, p = 0.003) and 5-year DSS (67.5% vs. 93.3%, p = 0.01) than the low-α-SMA group. RFS (p = 0.04) and DSS (p = 0.02) in the high-FAP group were significantly worse than those in the low-FAP group. Multivariable analyses found that high α-SMA expression was an independent predictor of RFS [hazard ratio (HR): 3.68; 95% confidence intervals (CI): 1.21–12.4; p = 0.02] and DSS (HR: 8.54; 95% CI: 1.21–170; p = 0.03).

**Conclusions:**

CAFs, particularly α-SMA, can be useful predictors of survival in patients undergoing radical resection for ampullary carcinomas.

## 1 Introduction

Ampullary carcinomas of the duodenum are rare neoplasms that arise in the ampulla of Vater. Surgical resection is a standard treatment for ampullary carcinoma, and relatively good prognosis has been reported after radical resection (1, 2). Histologically, ampullary carcinoma can be divided into three subtypes: intestinal, and pancreatobiliary, and mixed types (3, 4). As several studies have reported the better prognosis and non-invasive nature of the intestinal type, the prognostic role of the histological subtypes has been currently recognized (5). However, the morphology and immunohistochemical features of ampullary carcinomas have been poorly investigated, owing to its rarity (3, 4).

The tumor microenvironment or stroma is a multicellular system consisting of mesenchymal, endothelial, and hematopoietic cells in the extracellular matrix (6). Cancer-associated fibroblasts (CAFs) are key components of the tumor microenvironment with various functions, including cancer initiation and progression (7). Markers of fibroblast subtypes include α-smooth muscle actin (α-SMA) and fibroblast activation protein (FAP) (8). The clinical implications of CAFs as biomarkers and potential targets for prevention and treatment have been discussed in gastrointestinal oncology (9, 10). However, the role of CAFs in ampullary carcinoma has rarely been investigated.

This study aimed to investigate the presence of CAFs in patients with ampullary carcinoma. We also evaluated the effect of CAFs on the survival of patients with ampullary carcinoma.

## 2 Materials and methods

### 2.1 Patients

We retrospectively reviewed 78 patients with ampullary carcinoma who underwent pancreatoduodenectomy at our institution between January 2000 and December 2021. The study protocol was approved by the Institutional ethics committee (approval no. 2110-003), and was conducted in accordance with the Declaration of Helsinki.

### 2.2 Data extraction

Clinicopathological data were extracted from our database: age, sex (male or female), body mass index, American Society of Anesthesiologists (ASA) physical status (grade 1, 2, or 3), hypertension, diabetes mellitus, preoperative biliary drainage, operative time, estimated blood loss, postoperative outcome [major complication defined as Clavien grade ≥3 (11) and mortality), pathological factors evaluated by the General Rules for Clinical and Pathological Studies on Cancer of the Biliary Tract of Japan (12) (T and N factors), histopathologic subtype evaluated by a pathologist (intestinal, pancreatobiliary, and mixed type) (13), recurrence (absence or presence), status at the last follow-up (survival or death), and cause of death (primary disease-related or others).

### 2.3 Immunohistochemical analysis

We employed a previously reported protocol for evaluating CAFs (14–16). First, the presence of tumor cells was confirmed by hematoxylin and eosin (H&E) staining. Sections on the microslides were deparaffinized with xylene, hydrated using a diluted alcohol series, and immersed in H2O2 with methanol to quench endogenous peroxidase activity. To reduce nonspecific staining, each section was blocked with a serum-free protein block (Dako, Agilent Technologies, Santa Clara, CA, USA) for 15 min. After heat-mediated antigen retrieval with Tris/EDTA buffer, the sections were incubated with anti-SMA antibody (1:1000 dilution; Sigma-Aldrich, St. Louis, MO, USA) or anti-FAP antibody (1:250 dilution; Abcam, Cambridge, UK) diluted in Dako REAL Antibody Diluent (Dako, Agilent Technologies, Santa Clara, CA, USA) and incubated overnight at 4°C. The sections were then incubated with Envision+ anti-mouse/rabbit antibodies (Dako, Agilent Technologies, Santa Clara, CA, USA) for 30 min at RT. The chromogen used was liquid DAB+ (Dako, Agilent Technologies, Santa Clara, CA, USA). The sections were visualized with a 3,3’-diaminobenzidine tetrahydrochloride solution, and nuclei were counterstained with Meyer’s hematoxylin. CAFs were defined as spindle-shaped cells expressing α-SMA or FAP, and evaluated using an area index calculated in low-magnification fields using ImageJ software (http://rsb.info.nih.gov/ij/). Three fields, including stromal cells per sample, were carefully selected to evaluate CAFs. The mean value obtained from each sectioned tissue sample was defined as the area index. The α-SMA and FAP positive rates were calculated as each area index. In this study, the median values of the area index for α-SMA and FAP were used as cut-off values to define the low and high groups.

### 2.4 Endpoints

The primary endpoint was the prognostic factors for survival after surgery. The secondary endpoint was survival after surgery, focusing on CAFs. The recurrence-free (RFS) and disease-specific (DSS) survival rates were analyzed.

### 2.5 Statistics

RFS and DSS rates were investigated using the Kaplan–Meier method, and the log-rank test was used to evaluate differences between the groups. DSS was defined as the duration from surgery to the date of death as a result of the primary disease. Patients who died of causes unrelated to the primary disease were excluded.

The prognostic factors associated with RFS and DSS were investigated using a Cox proportional hazards model with hazard ratios (HR) and 95% confidence intervals (CI). As a significant correlation between α-SMA and FAP was found, the multivariable analyses were generated by including α-SMA and FAP separately. In Model 1, relevant factors, including α-SMA, were included in the multivariable analyses. In Model 2, the multivariable analyses included relevant factors, including FAP.

JMP version 11 software (SAS Institute, Cary, NC, USA) was used for statistical analyses.

## 3 Results

### 3.1 Study cohort

Of the 78 patients, 67 were available for immunohistochemical analysis. The characteristics of 67 patients are shown in **Table 1**. Pathological T factors included Tis (n = 10), T1 (n = 21), T2 (n = 22), and T3 (n = 17). Lymph node metastases were observed in 14 patients (21%). Histopathologic subtypes of ampullary carcinoma included the intestinal (n = 36, 54%), pancreatobiliary (n = 20, 30%), and mixed type (n = 11, 16%). Postoperative recurrence was observed in 17 patients (25%) during a mean follow-up period of 5.3 years.

**Table 1:** Clinicopathological characteristics of patients with ampullary carcinoma.

	Patients (n = 67)
Demographic variables	
Age (year)	70.3 (9.2)
Sex (male/female)	40 (60)/27 (40)
BMI (kg/m^2^)	22.0 (3.5)
ASA-PS (1–2/3)	56 (84)/11 (16)
Hypertension	27 (40)
Diabetes mellitus	19 (28)
Preoperative biliary drainage	35 (52)
Perioperative factors	
Operative time (min)	420 (87)
Blood loss (mL)	374 (347)
Major complication (presence/absence)	8 (13)/59 (87)
Mortality (presence/absence)	0 (0)/67 (100)
Pathological factors	
T factor (Tis/1/2/3)	7 (10)/21 (31)/22 (33)/17 (25)
Lymph node metastasis (presence/absence)	14 (21)/53 (79)
Histopathologic subtype	
Intestinal type	36 (54)
Pancreatobiliary type	20 (30)
Mixed type	11 (16)
Cancer-associated fibroblasts	
α-SMA (n = 66)	11.0 (10.5)
FAP	6.9 (9.6)
Recurrence (presence/absence)	17 (25)/50 (75)

Data are presented as mean (± standard deviation) or number (percentage).

BMI, body mass index; ASA-PS, American Society of Anesthesiologists physical status.

### 3.2 CAF expression

**Figure 1:**
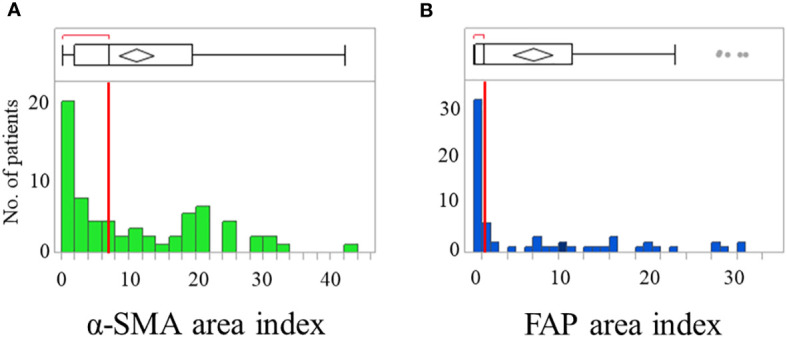
Distribution of patients showing α-smooth muscle actin (α-SMA) **(A)** and fibroblast activation protein (FAP) **(B)** area index. Box plots show median with the interquartile range; whiskers give the range.

**Figure 2:**
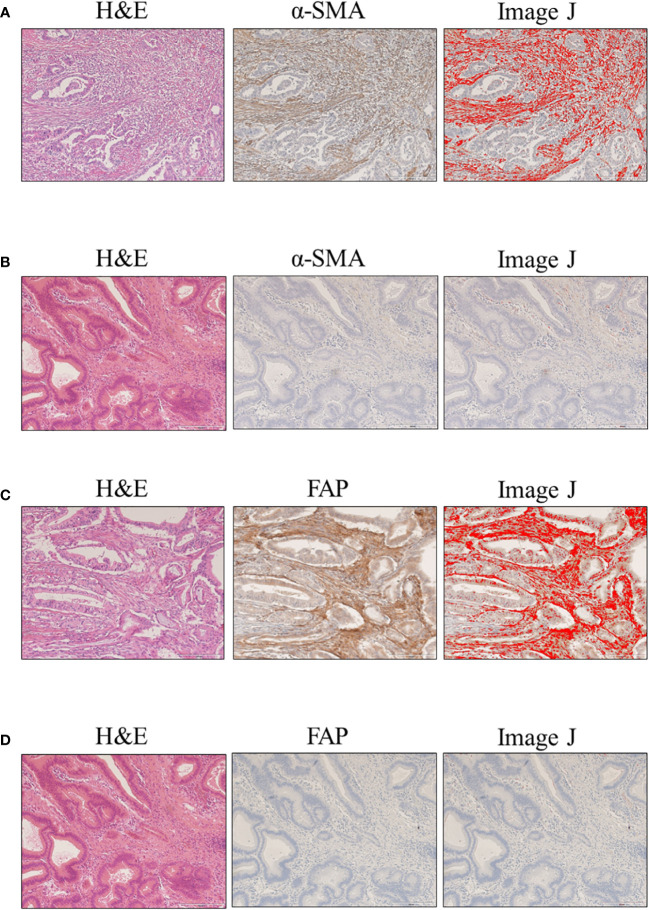
Evaluation of α-smooth muscle actin (α-SMA) and fibroblast activation protein (FAP) expression in clinical samples of ampullary carcinoma. Microscopic images with hematoxylin and eosin (H&E), cancer-associated fibroblasts (CAF) staining, and Image J: **(A)** high α-SMA expression; **(B)** low α-SMA expression; **(C)** high FAP expression; and **(D)** low FAP expression.

**Figure 3:**
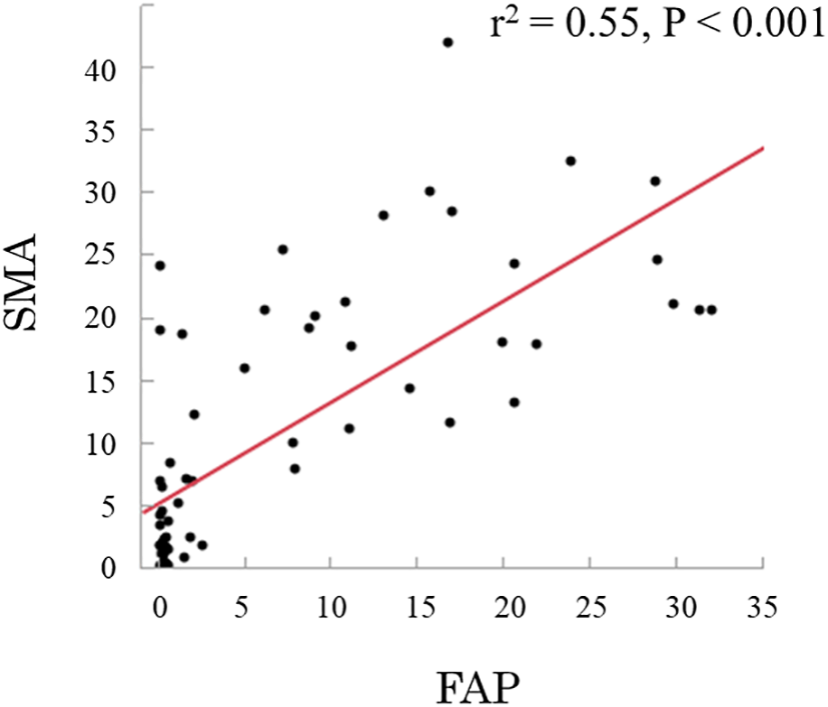
Relationship between smooth muscle actin (α-SMA) and fibroblast activation protein (FAP), showing a linear correlation (r^2 =^ 0.55; p < 0.001).

CAFs were identified as stromal cells expressing α-SMA and FAP. The mean (standard deviation [SD]) area indices for α-SMA and FAP were 11.0 (10.5) and 6.9 (9.6), respectively (**Figure 1**). Using the median area indices for α-SMA and FAP, the cut-off values were set at 7.1 for α-SMA and 1.2 for FAP. Microscopic images of H&E, anti-α-SMA, and FAP staining, as well as the images generated using Image J, are shown in **Figure 2**. The area index of α-SMA significantly correlated with that of FAP (r^2^ = 0.55; p < 0.001), as shown in **Figure 3**.

### 3.3 Association of CAF expression with histopathologic subtype

Relationship between CAF expression and histopathologic subtype of ampullary carcinoma is depicted in **Table 2**. A significant difference was found in the mean (SD) area index for α-SMA and FAP between three groups: 4.8 (5.9) and 2.2 (5.8) in the intestinal type; 19.1 (9.9) and 12.8 (10.1) in the pancreatobiliary type; and 16.0 (10.8) and 11.1 (11.0) in the mixed type. The high-α-SMA and high-FAP was 25% and 19% in the intestinal type, 90% and 80% in the pancreatobiliary type, and 73% and 91% in the mixed type, respectively.

**Table 2:** Association of CAF expression with histopathologic subtype of ampullary carcinoma.

	Intestinal type (n = 36)	Pancreatobiliary type (n = 20)	Mixed type (n = 11)	p-value
α-SMA (n = 66)	4.8 (5.9)	19.1 (9.9)	16.0 (10.8)	<0.001
Low	28	2	3	<0.001
High	7	18	8	
FAP	2.2 (5.8)	12.8 (10.1)	11.1 (11.0)	<0.001
Low	29	4	1	<0.001
High	7	16	10	

Data are presented as mean (± standard deviation) or number.

### 3.4 Association of CAF expression with survival

**Figure 4:**
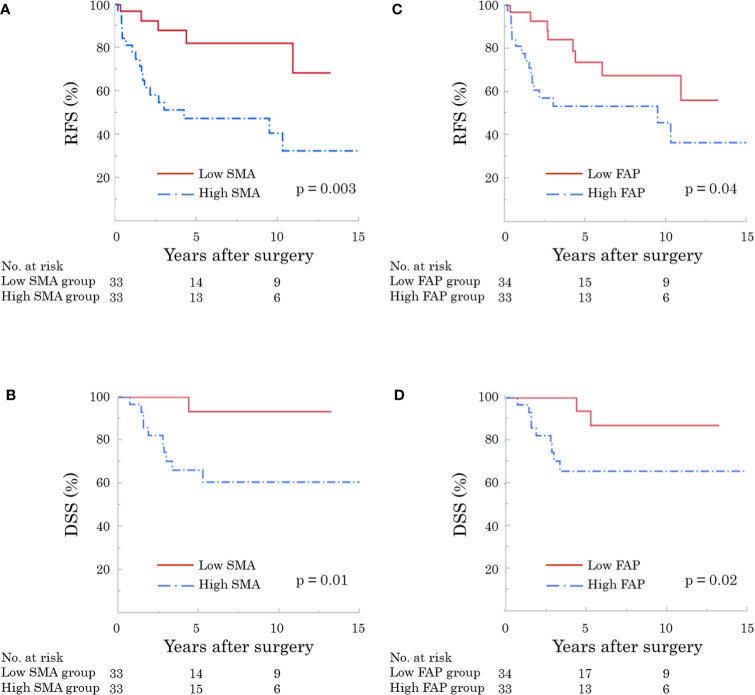
Recurrence-free (RFS) and disease-specific survival (DSS) according to expression of cancer-associated fibroblasts (CAF). The high α-smooth muscle actin (α-SMA) group had significantly worse RFS [**(A)** p = 0.003] and DSS [**(B)** p = 0.01]. In addition, patients with high fibroblast activation protein (FAP) showed significantly worse RFS [**(C)** p = 0.04] and DSS [**(D)** p = 0.02].

The 5-year RFS and DSS rates were 63.9% and 78.5%, respectively. RFS and DSS curves stratified by α-SMA and FAP are shown in **Figure 4**. Patients with high α-SMA expression had a significantly worse RFS than those with low α-SMA expression (5-year RFS, 47.6% vs. 82.2%; p = 0.003; **Figure 4A**). Furthermore, the 5-year DSS rates in the low- and high-α-SMA groups were 93.3% and 67.5%, respectively, with a significant difference between the groups (p = 0.01; **Figure 4B**). Regarding the effect of FAP on survival, the high-FAP group had significantly worse RFS (5-year RFS, 53.5% vs. 73.7%; p = 0.04; **Figure 4C**) and DSS (5-year DSS, 65.7% vs. 94.4%; p = 0.02; **Figure 4D**) than the low-FAP group.

### 3.5 Prognostic factors associated with survival

The results of the univariate and multivariable analyses for investigating the prognostic factors of RFS are shown in **Table 3**. Univariate analyses revealed that α-SMA, FAP, and lymph node metastasis were significant factors, but histopathologic subtypes were not an independent factor. In model 1, multivariable analysis revealed that high α-SMA expression was an independent predictor (HR: 3.68; 95% CI: 1.21–12.4; p = 0.02). In contrast, high FAP was not an independent index for RFS in model 2.

**Table 3:** Univariate and multivariable analyses of prognostic factors associated with recurrence-free survival.

	Univariate analysis			Multivariable analysis (Model 1)			Multivariable analysis (Model 2)		
Variable	HR	95% CI	p-value	HR	95% CI	p-value	HR	95% CI	p-value
αSMA									
Low	1								
High	3.94	1.57–12.0	0.003	3.68	1.21–12.4	0.02			
FAP									
Low	1						1		
High	2.31	1.01–5.69	0.047				2.03	0.75–5.57	0.15
Sex									
Female	1			1			1		
Male	1.39	0.61–3.44	0.44	2.09	0.86–5.49	0.11	2.00	0.82–5.29	0.13
Age (years)									
< 70	1								
≥ 70	1.85	0.81–4.58	0.14						
BMI (kg/m^2^)									
< 25	1								
≥ 25	0.50	0.08–1.71	0.30						
ASA-PS									
Grade 1–2	1								
Grade 3	1.52	0.44–4.06	0.47						
Major complication									
Absence	1								
Presence	1.50	0.43–4.02	0.48						
T factor									
< T3	1			1			1		
T3	1.65	0.67–3.79	0.27	0.81	0.29–2.10	0.67	0.98	0.37–2.45	0.97
Lymph node metastasis									
Absence	1			1			1		
Presence	3.23	1.34–7.42	0.01	2.38	0.87–6.34	0.09	2.88	1.11–7.25	0.03
Histopathologic subtype									
Intestinal type	1								
Pancreatobiliary type	2.75	1.10–6.97	0.03						
Mixed type	2.75	0.84–8.01	0.09						

HR, hazard ratio; CI, confidence interval; BMI, body mass index; ASA, American Society of Anesthesiologists.

**Table 4** shows the results of the univariate and multivariable analyses for DSS. Univariate analyses identified high α-SMA (HR: 9.48; p = 0.005) and FAP (HR: 4.50; p = 0.03) as independent predictors of DSS. However, only high α-SMA level (HR: 8.54; 95% CI: 1.21–170; p = 0.03) was a significant factor associated with DSS in the multivariable analyses.

**Table 4:** Univariate and multivariable analyses of prognostic factors associated with disease-specific survival.

	Univariate analysis			Multivariable analysis (Model 1)			Multivariable analysis (Model 2)		
Variable	HR	95% CI	p-value	HR	95% CI	p-value	HR	95% CI	p-value
αSMA									
Low	1			1					
High	9.48	1.81–174	0.005	8.54	1.21–170	0.03			
FAP									
Low	1						1		
High	4.50	1.16–29.6	0.03				2.92	0.57–22.1	0.21
Sex									
Female	1								
Male	2.01	0.58–9.19	0.28						
Age (years)									
< 70	1								
≥ 70	1.62	0.49–6.18	0.44						
BMI (kg/m^2^)									
< 25	1								
≥ 25	0.58	0.03–3.06	0.58						
ASA-PS									
Grade 1–2	1			1			1		
Grade 3	4.69	1.20–16.1	0.03	4.42	0.94–20.6	0.06	3.67	0.82–15.9	0.09
Major complication									
Absence	1								
Presence	2.16	0.47–7.52	0.29						
T factor									
< T3	1			1			1		
T3	2.14	0.62–7.11	0.22	0.56	0.12–2.42	0.44	0.79	0.19–3.16	0.74
Lymph node metastasis									
Absence	1			1			1		
Presence	2.55	0.67–8.45	0.16	1.59	0.40–5.68	0.49	1.92	0.47–7.12	0.34
Histopathologic subtype									
Intestinal type	1								
Pancreatobiliary type	4.50	1.18–21.4	0.03						
Mixed type	3.49	0.46–21.2	0.20						

HR, hazard ratio; CI, confidence interval; BMI, body mass index; ASA, American Society of Anesthesiologists.

### 3.6 CAF expression and clinicopathological parameters

The relationship between α-SMA expression and clinicopathological parameters is shown in **Table 5**. No significant differences were found between the low- and high-α-SMA groups in terms of the demographic variables. High α-SMA expression was significantly associated with advanced T stage as well as higher incidences of lymph node metastases and recurrence. In fact, the low α-SMA group had lymph node metastasis in only one patient (3%) and no recurrence after surgery.

**Table 5:** Relationship between αSMA expression and clinicopathological parameters.

	Low α-SMA (n = 33)	High α-SMA (n = 33)	p-value
Demographic variables			
Age (year)	69.9 (9.6)	70.8 (8.9)	0.74
Sex (male/female)	21 (64)/12 (36)	18 (55)/15 (45)	0.45
BMI (kg/m^2^)	22.6 (4.1)	21.5 (2.7)	0.14
ASA-PS (1–2/3)	29 (88)/4 (12)	23 (70)/7 (30)	0.32
Pathological factors			
T stage (Tis/1/2/3)	7 (21)/17 (52)/9 (27)/0 (0)	0 (0)/4 (12)/13 (39)/16 (48)	<0.001
Lymph node metastasis (presence/absence)	1 (3)/32 (97)	12 (36)/21 (64)	<0.001
Histopathologic subtype			
Intestinal type	28 (85)	7 (21)	<0.001
Pancreatobiliary type	2 (6)	18 (55)	
Mixed type	3 (9)	8 (24)	
Recurrence (presence/absence)	0 (0)/33 (100)	16 (48)/17 (52)	<0.001

Data are presented as mean (± standard deviation) or number (percentage).

BMI, body mass index; ASA-PS, American Society of Anesthesiologists Physical Status.

## 4 Discussion

This study is the first to investigate the significance of CAFs in patients undergoing pancreatoduodenectomy for ampullary carcinoma. We found CAFs in ampullary carcinoma. Furthermore, our results revealed that CAFs, especially α-SMA, are significantly associated with survival after radical resection.

Interesting association of CAF expression with histopathologic subtype of ampullary carcinoma was detected in this study. A novel finding included that the intestinal type was associated with a lower area index of α-SMA as well as FAP compared to those of the pancreatobiliary and mixed types (**Table 2**). Similar to previous reports (5), prognosis in the intestinal type was better than those in the pancreatobiliary and mixed types (**Supplementary Figure 1**). Moreover, multivariable analyses revealed that CAF expression was a stronger predictor of survival than histopathologic subtypes. Further research would be required to examine the interaction between CAF and histopathologic subtypes.

The present study reveals a strong relationship between CAFs and survival. Patients with high α-SMA and FAP expression had significantly worse RFS and DSS (**Figure 4**), in line with previous reports in gastrointestinal surgical oncology (14, 15). Moreover, our multivariable analyses suggested that α-SMA was an independent predictor of RFS and DSS after surgery (**Tables 3**, **4**). There was a significant association between α-SMA expression and pathological factors (**Table 5**). Based on the relationship between α-SMA expression and advanced tumor stages, the findings of multivariable analyses can be explained. Briefly, patients with high α-SMA expression had more advanced tumors and a higher incidence of lymph node metastases, leading to worse RFS and DSS.

The role of CAFs has recently gained widespread attention in the field of cancer biology. CAF biology is mediated through direct and paracrine interactions of cellular and acellular compartments (9). The role of CAFs in cancer invasion and metastasis has been investigated over the past few years. A recent review reported the association of CAFs with cancer invasion and metastasis that occurs through extracellular matrix deposition and remodeling, epithelial-mesenchymal transition in cancer cells, and secretion of growth factors supporting cancer cells (17). Furthermore, potential CAF-targeted therapeutic strategies have been suggested (6, 14–17). There are ongoing clinical trials investigating the efficacy of CAF-targeted therapies combined with existing therapies (8, 18–20).

Current extensive research has demonstrated subtypes of CAFs including pro-tumor or anti-tumor characteristics. Recent studies have supported the evidence for CAF heterogeneity in pancreatic ductal adenocarcinoma, showing several subpopulations of CAFs such as myofibroblastic CAFs (myCAFs), inflammatory CAFs (iCAFs), and antigen-presenting CAFs (apCAFs) (21, 22). However, the role of CAFs subtypes of ampullary carcinoma has not yet been investigated.

Translating the results of this study into clinical practice is important. The assessment of CAF expression can be easy and useful for detecting high-risk patients who could have poor long-term outcomes. Although the utility of adjuvant therapy for high-risk patients with ampullary carcinoma has been suggested (23), further studies are required to understand its biological features and histological characteristics and to develop an optimal therapeutic strategy to treat ampullary carcinoma (4). Therefore, evaluation of CAFs could be regarded as a novel treatment strategy in decision making for the introduction of adjuvant or first-line chemotherapy.

This study has several limitations, given that was a retrospective single-center study. The sample size was relatively small because of the rarity of the disease. Further studies with larger sample sizes are needed to clarify the role and efficacy of CAFs in ampullary carcinoma. The detailed mechanisms underlying the interaction between CAFs and prognosis are unknown. We suggest that CAFs promote epithelial-mesenchymal transition as well as cancer invasion and metastasis, including lymph node metastasis (17), leading to worse prognosis in patients with ampullary carcinoma. However, further studies should be performed to identify and delineate the interactions among CAFs, epithelial-mesenchymal transition, and cancer invasion.

## 5 Conclusion

The present study indicates that the assessment of CAFs can be helpful in evaluating cancer progression as well as in estimating survival after radical resection in patients with ampullary carcinoma.

## Data availability statement

The raw data supporting the conclusions of this article will be made available by the authors, without undue reservation.

## Ethics statement

The studies involving human participants were reviewed and approved by Okayama University Hospital. The ethics committee waived the requirement of written informed consent for participation.

## Author contributions

Concept and study design: KT, KN, and SK; Acquisition of data: KT, YN, YU, RY, TomF, KY, and HK; Pathological evaluation: TT; Drafting of the manuscript: KT; Critical revision of the manuscript for important intellectual content: KN, SK, YU, RY, TY and TosF. All authors contributed to the article and approved the final version of the article.

## Supplementary material

Supplementary Figure 1 Recurrence-free (RFS) and disease-specific survival (DSS) by histopathologic subtypes. The 5-year RFS showed 79.0% in the intestinal group, 43.6% in the pancreatobiliary group, and 53.9% in the mixed group (**A**; p = 0.046). The 5-year DSS included 91.8% in the intestinal group, 60.5% in the pancreatobiliary group, and 71.4% in the mixed group (**B**; p = 0.07).
