# Supplementary figures and images for: Impact of cancer-associated fibroblasts on survival of patients with ampullary carcinoma
Source: Front Oncol. 2023 Mar 16;13:1072106. doi: 10.3389/fonc.2023.1072106 (PMC10060636; doi:10.3389/fonc.2023.1072106)

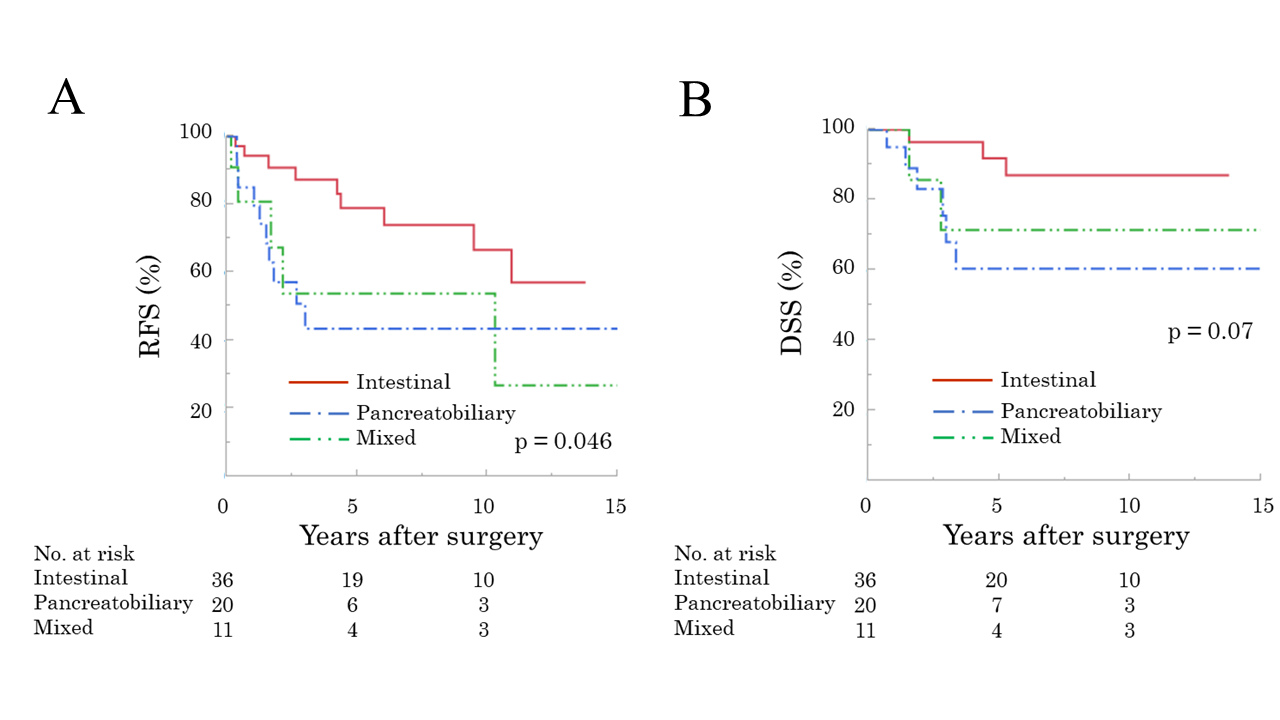

Supplement: Supplementary Figure 1 — Recurrence-free (RFS) and disease-specific survival (DSS) by histopathologic subtypes. The 5-year RFS showed 79.0% in the intestinal group, 43.6% in the pancreatobiliary group, and 53.9% in the mixed group (A; p = 0.046). The 5-year DSS included 91.8% in the intestinal group, 60.5% in the pancreatobiliary group, and 71.4% in the mixed group (B; p = 0.07). [file Image_1.tif]
